# Philadelphia Hospital

**Published:** 1838-02-28

**Authors:** 


### PHILADELPHIA HOSPITAL.

*Saturday, 24th February.* At half past eleven, Dr. GIBSON appeared in the amphitheatre, having, as he remarked, obtained the use of the lecture-room, for a few minutes, from Dr. Jackson. You may remember, gentlemen, said he, that upon taking leave of you, sometime since, I promised to retain two or three interesting cases and present them to your notice, at a future period. The two cases I now show you are those of ulcerated lip and osteo-sarcomatous jaw. With the history of both you are well acquainted. I first call your attention to the ulcerated lip. You see a decided and evident amendment, produced by the constitutional remedy, administered some weeks ago, and continued up to this time, viz. Marks' Extract of Sarsaparilla, and also by the local application of chloride of zinc. The sore has nearly healed, and the portion of the ulcer, confined to the lower lip, entirely closed.

I next call your attention, gentlemen, to another theme,—the osteo-sarcomatous jaw, and certain circumstances connected with it. I deeply regret, that I had not the good fortune to listen to the *introductory* lecture of my distinguished colleague, whose interesting *valedictory* you have just heard, with so much patience and pleasure, whose acknowledged talent, and whose brilliant display of eloquence have enchained your attention, for the last few weeks. Could it be otherwise, than that such a voice, loud and sonorous, peculiarly musical in its tone, clear and delightful in its cadence, should fall upon your ear, with all the harmony of sympathetic association? We feel peculiarly happy, gentlemen, in bearing testimony to the merits of our colleague, and have no doubt that the managers have retained his important services, in this great establishment, for the purpose of attracting to it full classes, such as I have the honour to be surrounded by, at this moment,—well knowing that his fine elocution, great experience as a surgeon, extraordinary intellectual endowments, and most amiable disposition, could not fail to produce such a result. Not only, indeed, is he distinguished in his particular vocation, but he has decided advantages over his less fortunate brethren, in being acknowledged, both in this country and in Europe, as profoundly acquainted with natural philosophy, natural history, in all its branches, and, more especially, with ornithology, zoology, conchology, entymology, astrology and demonology.

That he stands unrivalled as an ornithologist, on this side of the Atlantic, is abundantly proved by the fact, that the celebrated Audubon has named the finest bird that ever sailed over the tops of the lofty forests of our western wilds—the "Falco Harlanus, or Black Warrior,"—after our distinguished colleague and friend. His large and splendid volume, too, on newts and frogs, toads and beetles, lizards and snakes, intermingled, most naturally, with aneurisms and tumours, fistulæ and strictures, written, indeed, "*de omnibus rebus et quibusdam aliis,*" affords additional testimony of his industry and talents. I sincerly hope that the gentleman is not at this moment snugly ensconsed behind the barriers of the upper benches; for knowing, as I well do, his extreme modesty, meekness and humility, his tender sensibilities could not fail, I am sure, to be shocked by praise of this public description.

After thus acknowledging, gentlemen, the merits of our accomplished colleague, need I, as an atonement for error, tell you, in the language of O'Connell, that you have been "cheated, and humbugged, and bamboozled," by Drs. Horner, Pancoast, and myself; that, from ignorance, and want of anatomical and surgical knowledge, we have committed a flagrant outrage, and performed a most severe and cruel operation upon the unfortunate human being, now before you; that we have used horrid, crooked knives, gouges, chisels, and mallets, and saws, and used them unrelentingly and ferociously, and that we have even applied the flaming cautery to the delicate and sensitive alveolar processes, and to the enamel of the teeth, while he himself would not even touch with it, so insensible a structure as the human eye or eye-lid. You will remember, no doubt, in how awkward a manner, I used these ungainly implements, and how near I came, by this awkwardness, to singing the eye-brows of my worthy colleague, while he was scientifically exploring the cells and caverns of the tumour, as I quarried it from its bed. You see that we three culprits have confounded this malignant fungus of the antrum, with osteo-sarcoma of the jaw. You see that we have all been mistaken in our diagnosis, and, in proof of it, you see, in the unfortunate being before you, how the tumour, as large as a cocoa-nut, projects beyond the face, how the eye is squeezed out of its socket, how the mouth is filled, and the patient's breathing embarrassed by a voluminous fungus from the antrum and the throat, how he is reduced to a skeleton, and so weak, that he can not sit upright on the chair, which possibly he will never leave alive.

Knowing all these things then, was it not kind, very kind, on the part of our amiable and humane colleague, to visit privately this poor and friendless being, and remonstrate with the house physicians against our proposed operation, and not only so, but, in the pure spirit of philanthrophy and humanity, to appeal to the better feelings of the managers themselves, and put a stop to such contemplated atrocities. That a stop was put to them, for a time, you also know, and well had it been for us all, for the honor of this great house, and for the credit of human nature, that they had been stopped altogether. But it was far otherwise, and it shall be the business of our lives, gentlemen, to atone for the manifold sins we have committed. All that we ask is forgiveness of these sins, and, under a promise of doing better in future, that our "Warrior Eagle," in all the nobleness of his nature, will still "suffer little birds to sing."

